# Effectiveness of Tap Water in Reducing the Generation of Ultrafine Wear Particles from the Wheel-Rail Contact by Eliminating the Water Vapor Effect

**DOI:** 10.3390/toxics11100873

**Published:** 2023-10-20

**Authors:** HyunWook Lee

**Affiliations:** 1 Transportation Environmental Research Department, New Transportation Innovative Research Center, Korea Railroad Research Institute, 176 Cheoldobangmulgwan-ro, Uiwang-si 16105, Gyeonggi-do, Republic of Korea; hwlee@krri.re.kr; Tel.: +82-31-460-5690; Fax: +82-31-460-5279; 2 Transportation System Engineering, University of Science Technology, 176 Cheoldobangmulgwan-ro, Uiwang-si 16105, Gyeonggi-do, Republic of Korea

**Keywords:** airborne wear particles, ultrafine particles, wheel-rail contact, tap water, diffusion dryer, subway system, wear

## Abstract

This study aimed to assess the impact of tap water application on reducing the generation of ultrafine particles from the wheel-rail contact using a twin-disk rig under dry and wet conditions, with train velocities of 45 and 80 km/h. A small amount of 0.3 L/min tap water was applied at the wheel-rail contact, and a diffusion dryer was used to eliminate water vapor. The Fast Mobility Particle Sizer measured the number concentration (NC) of nano-sized wear particles in the range of 6 to 560 nm. The tap water application method effectively reduced the NC of ultrafine and fine particles by 67–72% and 86–88%, respectively. Positive reduction rates were observed for all diameters at 45 km/h and for most diameters, except for approximately 70 nm and 80 nm, at 80 km/h. Even with a small amount of water, this approach successfully decreased nano-sized wear particle generation. However, the potential influence of mineral crystals in tap water on NC requires further investigation. Overall, this method shows promise for enhancing air quality and public health by mitigating nano-sized wear particle generation in subway systems.

## 1. Introduction

The subway system is generally considered an eco–friendly mode of transportation due to its use of electric power for train operations. However, studies conducted by Lee [1,2] have shown that subway trains continuously generate airborne wear particles (AWPs) from the contact between the wheel and rail. These AWPs constitute the primary source of AWPs within the subway system. The presence of AWPs in the subway system contributes to the degradation of air quality and poses potential risks to public health when inhaled [3,4,5,6,7,8,9,10,11,12].

The AWPs generated from the contact between the wheel and rail consist of various metal elements, including Cu, Mn, and Fe [4,13,14,15,16,17,18,19]. Smaller particles pose a greater risk as they are easily inhaled. Among them, ultrafine particles are particularly hazardous since they can travel through the blood stream, accumulate in human organs, and cause damage [6,8,20,21]. Therefore, it is crucial to minimize the generation of nano-sized wear particles, such as ultrafine and fine wear particles, at the wheel-rail contact in order to safeguard public health.

In the aspect of wear, wear particles are typically classified into three categories based on their size: ultrafine (particle diameter, *D_p_* ≤ 100 nm), fine (100 nm < *D_p_* ≤ 1 μm), and coarse (1 < *D_p_* ≤ 10 μm) particles. Coarse particles are mainly generated through mechanical wear processes, while ultrafine and fine particles are mainly generated through thermal wear processes, according to Zimmer and Maynard [22]. However, studies on AWPs generated from the wheel-rail contact are still in their early stage. Most studies [1,2,23,24,25,26,27] have focused on identifying the generation phenomena through field tests and determining the conditions and parameters affecting AWP generation under dry conditions. Studies on methods to reduce AWPs generated from the wheel-rail contact are extremely rare. Abbasi et al. [28] conducted tests using a pin–on–disk machine and reported that a water–based friction modifier reduced the number concentration (NC) of APWs, but this test was limited to pure sliding contact conditions. Lee [29], in a twin–disk rig that can simulate both rolling–sliding and pure sliding contacts, found that applying tap water at the wheel-rail contact reduced the NC of AWPs under different loads. Lee [30] further reported that applying tap water at the wheel-rail contact significantly decreased the NC of fine wear particles and reduced micro–sized wear particles to nearly negligible levels across various train velocities. However, Lee’s studies [29,30,31] were conducted under tap water conditions, showing an increase in particle NC of ≤35 nm in diameter, possibly due to water vapor and mineral crystals from tap water. However, these studies by Lee [29,30,31] did not utilize any form of dryer to remove water vapor generated at high contact temperature.

As a continuation of the previous study [31], this research aimed to verify the effectiveness of tap water application in reducing the NC of nano-sized wear particles generation, especially ultrafine particles, by eliminating the influence of water vapor. To ensure an accurate assessment of the reduction in nano-sized wear particle generation at the wheel-rail contact, a diffusion dryer was employed to eliminate the effect of water vapor.

## 2. Materials and Methods

### 2.1. Experimental Setup, Particle Sensor, Sample Disk

In this study, a newly developed small–sized twin–disk rig was utilized to investigate the generation of AWPs at the wheel-rail contacts. This rig was designed based on the twin–disk rig used in previous studies [1,2,27,29,30,31]. Each disk was connected to a-45 kW AC motor and driven independently. To measure the driving torque of the wheel and rail disks, separate torque cells were employed. Additionally, encoders attached to each motor were used to measure the rotational speed of each disk. The application of a radial force through a small motor enabled the generation of a normal contact force, which was measured using a load cell. The rotational speed of each disk was controlled independently using a PID controller.

**Figure 1:**
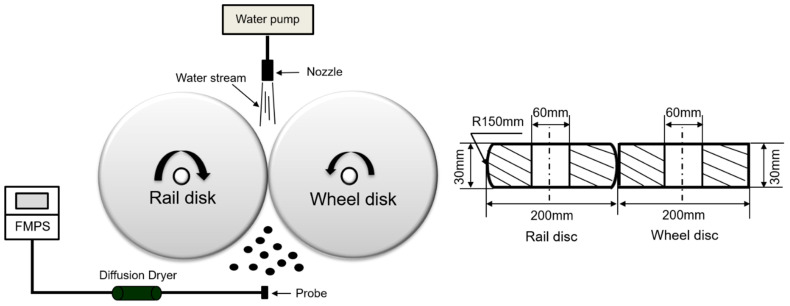
The schematic of the experimental setup and sample disks.

The experimental setup used to simulate the generation of AWPs at the wheel-rail contacts was described comprehensively in previous studies by Lee [1,2,27,29,30,31]. The schematic of the experimental setup is shown in Figure 1. Since this study focused on nano-sized wear particles, the Fast Mobility Particle Sizer (FMPS; TSI Inc., Shoreview, MN, USA) was used to measure the NC of ultrafine and fine particles generated at the wheel-rail contact. The FMPS can measure particle diameter ranging from 5.6 to 560 nm and operated at a sampling frequency of 1 Hz. To eliminate water vapor produced during the application of water, a diffusion dryer, as depicted in Figure 1, was connected between the FMPS and the probe. The diffusion dryer, measuring 250 mm in length and 55 mm in diameter, was filled with silica gel.

The chemical composition and hardness details for the wheel and rail disks are presented in Table 1. The wheel and rail disks underwent a heat treatment process to attain the desired levels of hardness, mirroring real-world conditions in the railroad system. The hardness of the disks in this study falls within the typical range observed in actual wheels and rails, thus ensuring that findings from this study are representative of the real-world scenario.

Schematic representations of the wheel and rail disks can be found in Figure 1. Both disks had a diameter of 200 mm and a thickness of 30 mm. The wheel disk had a flat tread, while the rail disk featured a profile head with a radius of 150 mm. The initial roughness (*R_a_*) values for the wheel and rail disks were measured to be 0.13 ± 0.02 μm and 0.17 ± 0.03 μm, respectively.

### 2.2. Test Conditions

In this study, subway train velocities of 45 km/h and 80 km/h were investigated, corresponding to rotational speeds of 1200 rpm and 2100 rpm, respectively. The choice of 80 km/h reflects the average velocity of subway train operations in Republic of Korea, while 45 km/h was selected from previous studies [1,27] to cover a wider range of subway train operational velocities, especially within the slower range. A normal contact force of 1470 N was applied, resulting in a maximum Hertzian contact pressure of 1060 MPa, representing the contact between a new wheel and rail. The study employed a slip rate range of 0–5% for both dry and wet (applying tap water) conditions, covering pure rolling, rolling–sliding, and pure sliding contact conditions. For wet conditions, a water pump applied 0.3 L/min of tap water stream to the wheel-rail contact point, as illustrated in Figure 1.

### 2.3. Test Procedure

As a continuation of the previous study [31], the research closely followed the test procedure outlined therein. The procedure can be summarized as follows:

1. Background particle NC was measured for 20 s (background zone).
2. The rotational speeds of the disks were accelerated over 30 s to reach the target speeds of 1200 and 2100 rpm (acceleration zone).
3. The desired slip rate of 5% was achieved by maintaining the target rotational speed of the rail disk while linearly increasing the rotational speed of the wheel disk for 120 s (slip zone). Slip rate was calculated using Equation (1):

(1) $$Slip\;rate\left(\%\right)=\frac{R_{w}-R_{r}}{R_{r}}\times 100$$

where *R_w_* and *R_r_* represent the rotational speed of the wheel and rail disks, respectively.

4. Finally, the rotational speeds of both disks were decelerated to stop over 45 s (deceleration zone).

### 2.4. Data Analysis

**Figure 2:**
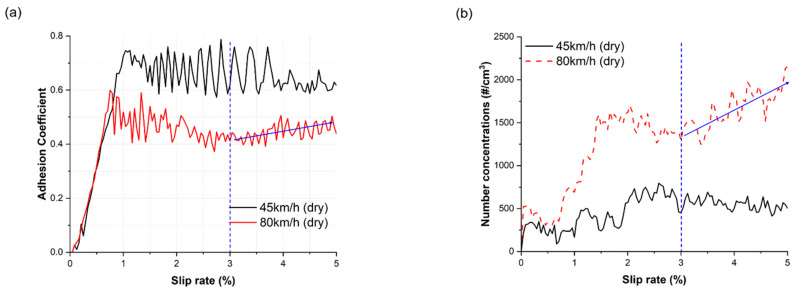
Adhesion coefficient and NC of fine particles at train velocities of 45 and 80 km/h as a function of the slip rate: (**a**) adhesion coefficient under dry conditions and (**b**) NC of fine particles under dry conditions. Arrows represent the unusual trend.

To assess particle formation at the wheel-rail contacts under dry and wet conditions, only the particle NC measured in the slip zone were considered. The adhesion coefficient (AC) for 2100 rpm showed an unusual trend after the 3% slip rate (Figure 2), distinct from the AC for 1200 rpm under dry conditions. This suggests a different contact condition after the 3% slip rate at 2100 rpm. Therefore, particle NC data in the slip rate range of 0–3% for both contact conditions were used for analysis in this study.

Two test trials were conducted for each test condition in this study [1,2,27,28,29,30,31,32]. For each test trial, the mean particle NC measured in the background zone was subtracted from the measurements taken in the slip rate range of 0–3% for each diameter range, eliminating the influence of the background particle NC. The generation characteristics of ultrafine and fine particles, along with the particle size number distribution (PSND) of total particles measured at each test condition, were examined across the entire slip rate range. Additionally, the reduction rate of ultrafine, fine, and total particles was calculated to verify the effectiveness of applying tap water, with the water vapor being removed using the diffusion dryer, in reducing the generation of nano-sized wear particles. The reduction rate was determined using the following Equation (2):

(2) $$Reduction\;rate=\left(\frac{C_{dry}-C_{wet}}{C_{dry}}\right)\times 100$$

## 3. Results

**Figure 3:**
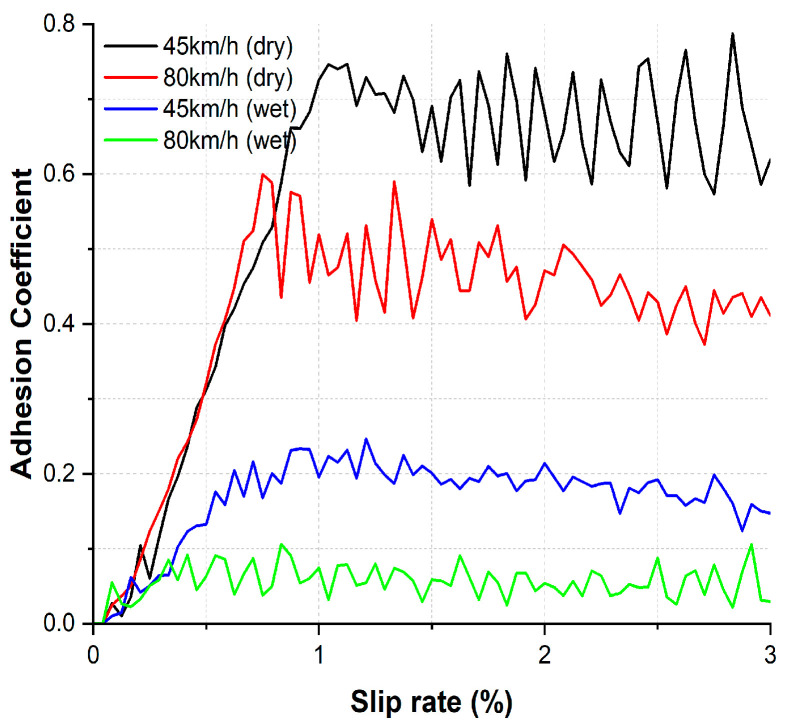
Representative ACs for two tested train velocities as a function of the slip rate under dry and wet conditions.

The ACs as a function of the slip rate under dry and wet conditions are presented in Figure 3 for the two tested train velocities. In both contact conditions, the ACs exhibited a decreasing trend after reaching its the maximum value. Notably, the AC at a train velocity of 45 km/h was higher than that at a velocity of 80 km/h under both dry and wet conditions. Furthermore, the AC under wet conditions were generally lower compared to the AC under dry conditions.

**Figure 4:**
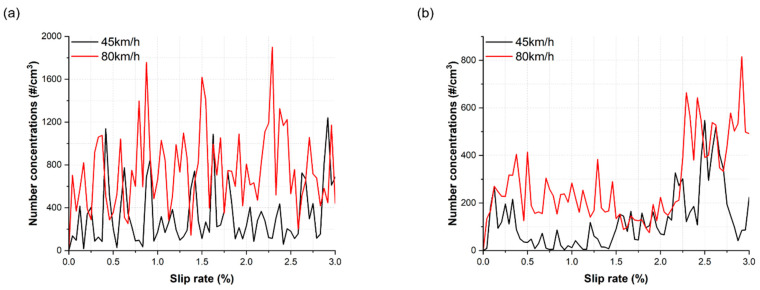

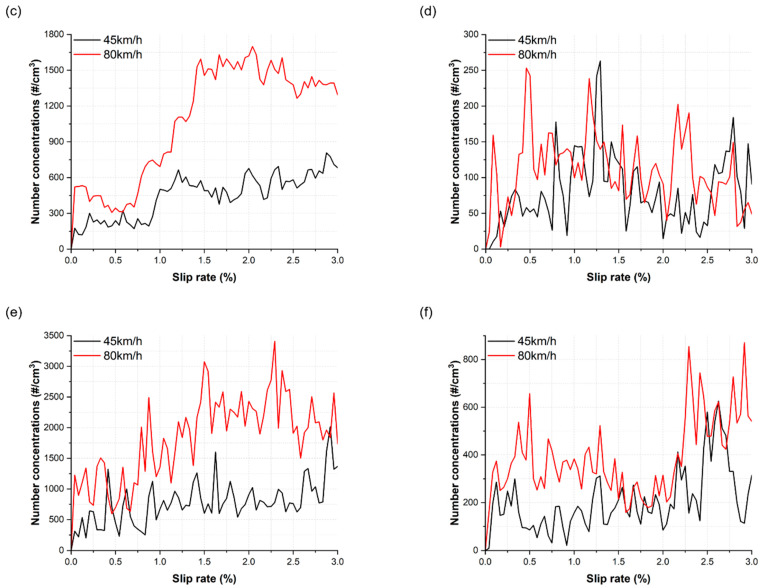
Representative NC for two tested train velocities as a function of the slip rate: (**a**) ultrafine particles under dry conditions, (**b**) ultrafine particles under wet conditions, (**c**) fine particles under dry conditions, (**d**) fine particles under wet conditions, (**e**) total particles under dry conditions, and (**f**) total particles under wet conditions.

The representative generation trends of the NC of ultrafine, fine, and total particles under dry and wet conditions are illustrated in Figure 4, as a function of the slip rate. Under dry conditions, at a train velocity of 45 km/h, the NC of ultrafine, fine, and total particles showed an increasing trend with the slip rate. At a train velocity of 80 km/h, the NC of ultrafine particles also exhibited a gradual increase, while the NC of fine and total particles showed an increasing–decreasing trend with the slip rate. Under wet conditions, the NC of ultrafine particles displayed a gradual increase for both velocities. However, the NC of fine particles increased with the slip rate up to approximately 1.25%, and beyond that point, they gradually decreased with further increases in the slip rate for both train velocities. In general, the NC of ultrafine and fine particles were higher at a velocity of 80 km/h compared to a velocity of 45 km/h under both dry and wet conditions.

**Figure 5:**
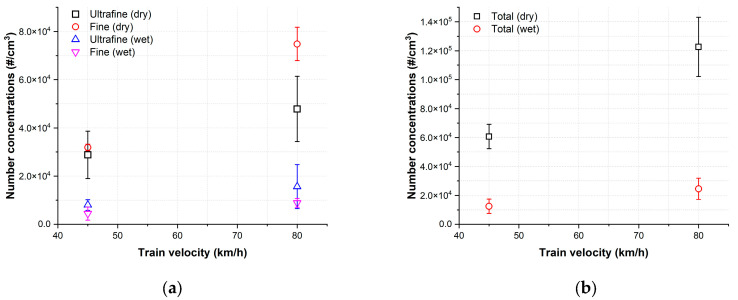
The mean and range of the sum of the measured NC under dry and wet conditions for each train velocity: (**a**) ultrafine and fine particles and (**b**) total NC. Error bars represent the range.

The sum of NC of ultrafine, fine, and total particles measured under dry and wet conditions at each train velocity is depicted in Figure 5. Under dry conditions, at a train velocity of 80 km/h, the NC of ultrafine, fine, and total particles were 1.7, 2.3, and 2.0 times higher than those at 45 km/h, respectively. Similarly, under wet conditions, the NC of ultrafine, fine, and total particles at a train velocity of 80 km/h were 1.9, 2.0, and 2.0 times higher than those at 45 km/h, respectively. Notably, the NC of both particle categories were significantly higher under dry conditions compared to wet conditions. Specifically, under dry conditions, the NC of fine particles were consistently higher than the NC of ultrafine particles for both train velocities. However, under wet conditions, this result was reversed.

The average reduction rates of the NC for ultrafine, fine, and total particles are summarized in Table 2. It is evident that the reduction rates of fine particles were higher than those of ultrafine particles for both train velocities. At both velocities, the reduction rates exceeded 67% for ultrafine particles and 85% for fine particles. Additionally, the reduction rate of total particles approached 80% for both train velocities.

**Figure 6:**
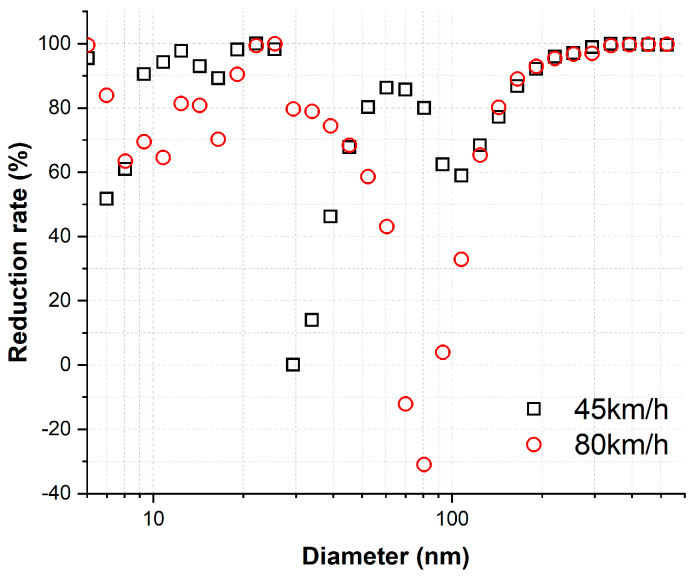
Average reduction rate of each particle diameter at each train velocity.

The average reduction rate of each particle diameter at each train velocity are illustrated in Figure 6. A positive value indicates that the NC of particles was higher under dry conditions than wet conditions. At a train velocity of 45 km/h, the reduction rates were positive for all particle diameters. At a train velocity of 80 km/h, the reduction rates were positive for most particle diameters, except for approximately 70 nm and 80 nm in diameter. It is evident that the reduction rates were predominantly greater than 50% for each diameter at both train velocities.

**Figure 7:**
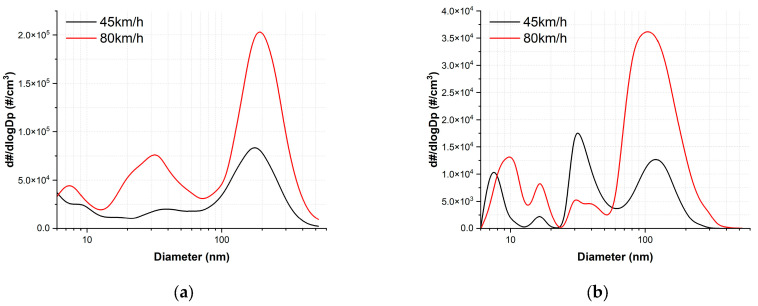
Representative PSNDs of total particles measured at the slip zone at each train velocity under (**a**) dry and (**b**) wet conditions.

The representative PSNDs of total particles at the two tested train velocities under dry and wet conditions are presented in Figure 7. Under dry conditions, the PSNDs exhibited a trimodal shape for both velocities, with peaks observed at approximately 10, 40, and 170 nm at 45 km/h, and at approximately 7, 35, and 190 nm at 80 km/h. Notably, the predominant peaks appeared in the fine particle category for both velocities, and the diameter of these peaks increased from approximately 170 to 190 nm with increasing train velocity.

Under wet conditions, the representative PSNDs displayed a multimodal shape with peaks observed at approximately 7, 17, 30, and 125 nm for 45 km/h, and at approximately 10, 17, 30, and 110 nm for 80 km/h. The predominant peak occurred at approximately 30 nm for 45 km/h and at approximately 110 nm for 80 km/h. The diameter of the predominant peak increased with train velocity and shifted from the ultrafine to fine particle category. Additionally, the particle NC at approximately 30 nm was considerably higher at 45 km/h compared to 80 km/h.

## 4. Discussion

In this study, a small–sized twin–disk rig was utilized to simulate the generation of AWPs at the wheel-rail contact under both dry and wet conditions, with train velocities of 45 km/h and 80 km/h. The twin–disk rig, designed for fundamental investigations into AWPs generation, is equipped with a 45 kW motor, which is lower in capacity than the 350 kW motors used in previous studies [1,2,27,29,30,31]. Due to this limitation, the maximum Hertzian contact pressure was set to 1000 MPa, which contrasts with the 1200 MPa used in earlier research. A maximum Hertzian contact pressure exceeding 1000 MPa is typically observed in the contact between new wheels and new rails.

In this study, the analysis focused on particle NC measured during the slip rate range of 0–3%. This decision was prompted by an unusual trend observed in the AC measured at 80 km/h, where it unexpectedly increased after a 3% slip rate, likely due to the high contact temperature [33,34,35]. At 45 km/h, the AC exhibited a decreasing trend after its peak value with increase slip rate, but at 80 km/h, the AC initially decreased up to 3% slip rate after its peak value and then increased up to 5%, as depicted in Figure 1. This unusual behavior is commonly associated with the presence of friction modifiers, like sand, on the rail surface, leading to an increase in AC [36]. It is possible that worn debris generated from the wheel-rail contact at higher slip rates accumulated and acted as friction modifiers, causing the increase in AC after 3% slip rate at 80 km/h. Consequently, the NC data up to a slip rate of 3% were considered for analysis in this study.

Under both dry and wet conditions, the measured AC displayed lower values at higher train velocity, and AC saturation was observed at approximately at a slip rate of 1% (Figure 3) [37,38,39]. The AC values recorded under wet conditions were notably lower compared to those under dry conditions. This difference can be attributed to the formation of a lubricating layer on the wheel-rail contact surface due to the presence of water, effectively reducing friction. The observed generation characteristics of the AC align well with findings from other studies [37,40].

The generation characteristics of ultrafine and fine particles substantiate the influence of train velocity on AWPs generation under dry conditions [1,27,30,31]. Notably, a higher NC of ultrafine and fine particles were generated at a train velocity of 80 km/h compared to 45 km/h (Figure 4a,c and Figure 5). As the slip rate increased, ultrafine particles displayed a gradual increasing trend at both train velocities, while fine particles exhibited an increasing–decreasing trend at 80 km/h and a consistent increasing trend at 45 km/h under dry conditions. Despite the prevalence of fine particles at both train velocities, the average proportion of fine particles in the total measured particles increased from 52.5% to 61% with the increase in train velocity. Regarding the NC, the diameter of the predominant peak and the particle diameter at the predominant peak both increased with train velocity, rising from 170 nm to 190 nm (Figure 7a).

The generation characteristics of ultrafine and fine particles were also influenced by the train velocity. The sum of ultrafine and fine particles generated across the entire slip range increased with train velocity (Figure 5). Additionally, as the train velocity increased, the NC at the peak diameters also showed an increase, and the diameter at the predominant peak shifted from 30 to 110 nm (Figure 7b). Ultrafine particles were predominantly generated at both train velocities, with the proportion of ultrafine particles showing a slight decrease from 64.2 to 63.7% as train velocity increased.

Applying water altered the generation characteristics in both particle categories. At both train velocities, in contrast to dry conditions, the ultrafine particles exhibited an irregular generation trend, while the fine particles demonstrated an increasing trend up to approximately 1.25% slip rate, followed by a decrease with further increments in slip rate (Figure 4b,d). Notably, under wet conditions, the predominant peak diameters were considerably smaller than those observed under dry conditions. Moreover, the peak diameter shifted from the category of ultrafine to fine particles with increasing train velocity, whereas they remained within the category of fine particles (Figure 7). Furthermore, the PSND results under wet conditions showed a multimodal shape at both train velocities, whereas those under dry conditions exhibited a trimodal shape (Figure 7). The total NC of ultrafine particles was higher than that of fine particles at both train velocities (Figure 5a). Additionally, the proportion of ultrafine particles remained higher than 63% at both velocities.

Under dry conditions, as shown Figure 5a, the NC of fine particles consistently exceeded that of ultrafine particles for both train velocities. However, under wet conditions, this trend was reversed. Under dry conditions, local combustion at the wheel-rail contact surface initiates evaporation when the frictional temperature surpasses a specific threshold. Subsequently, vapor is released from the friction surface, encounters the surrounding air, and rapidly cools, leading to the formation of ultrafine particles through nucleation. These newly generated particles become highly concentrated and grow through coagulation, forming agglomerates, or the vapor directly condenses onto the surfaces of existing particles. Under dry conditions, particle generation and enlargement occur through these processes. Conversely, under wet conditions, the application of water may play a pivotal role by reducing the frictional temperature of the contact surfaces, thereby lowering the probability of combustion and subsequently reducing the generation of material vapor. Consequently, the number of ultrafine particles produced through nucleation could be significantly diminished, leading to a decreased likelihood of particle size enlargement.

In the previous study [31], the presence of water vapor, generated by the high frictional heat at the wheel-rail contact, led to negative reduction rates at diameters below approximately 35 nm for all tested train velocities. To mitigate this water vapor effect, a diffusion dryer was employed in this study. The results demonstrated that reduction rates were positive for all measured diameters at 45 km/h and for most diameters, except approximately 70 and 80 nm, at 80 km/h. As a result, the findings of the reduction rate in this study validate that the generated water vapor has a substantial impact on the NC of ultrafine particles when AWP generation tests are conducted under wet conditions without employing a device to remove water vapor.

In the previous study [31], the possibility of a negative reduction rate observed in particles between approximately 10 and 35 nm in diameter was attributed to the presence of mineral crystals in the tap water used. In the current study, where tap water was also utilized, a low positive reduction rate of less than 50% was found only in the particle size range of 30–50 nm at 45 km/h (Figure 6). Despite implementing a diffusion dryer to remove water vapor generated by high frictional heat, it is important to note that mineral crystals are not absorbed or filtered out by the diffusion dryer. Consequently, particles with diameters approximately in the range of 30–50 nm could potentially be influenced by mineral crystals presented in the tap water. To further investigate this potential influence of mineral crystals, future studies should consider using distilled water instead of tap water. This change would help verify whether mineral crystals have any impact on the measured NC of ultrafine particles under wet conditions.

In previous studies [30,31], a fixed rate of 7 L/min of tap water was applied due to limitations of the available water application device. Consequently, this fixed rate was used in conducting those studies, leading to significant reductions in particle sizes larger than 100 nm. However, it should be noted that high water application rates could potentially result in a reduction of the adhesion coefficient and the introduction of a risk of derailment. In the current study, a newly developed water application device that allows for adjustable water volume was implemented, with a minimum setting of 0.3 L/min. Therefore, 0.3 L/min of tap water was applied to investigate its effectiveness in maintaining a relatively high adhesion coefficient while reducing the number concentrations of nano-sized wear particles. Tap water was chosen for this study because it has been employed in previous studies and is readily available everywhere.

This study employed a method to eliminate the effect of water vapor on the measured NC to verify the effectiveness of the water application in reducing ultrafine particles generated from the wheel-rail contact. The results revealed reduction rates of 86–88% for fine particles and 67–72% for ultrafine particles, observed at both train velocities. Nano-sized wear particles are mainly generated through the thermal wear process [22]. When water is applied, it forms a water film at the contact interface, serving as boundary lubrication. This water film leads to a reduction in wheel asperity–rail asperity contacts, thereby decreasing the frictional force and frictional heat. Additionally, the water film absorbs the frictional heat and carries it away from the contact interface, effectively lowering the contact temperature. As consequence, the water application method prevents the conditions necessary for the generation of nano-sized wear particles, ultimately reducing their generation. Based on the study’s findings, it can be inferred that the water application method is indeed effective in reducing the generation of ultrafine particles by eliminating the water vapor.

## 5. Conclusions

The purpose of this study was to validate that applied tap water can effectively reduce the NC of ultrafine particles by eliminating the water vapor effect, building upon the findings of a prior study [31]. As part of this consecutive investigation, the study examined the generation of ultrafine and fine particles under both dry and wet conditions at two operational velocities of 45 and 80 km/h, using a diffusion dryer. The NC of ultrafine and fine particles and the PSNDs of the total particles were analyzed and compared at each train velocity under dry and wet conditions. It is important to note that in this study, a very small amount of tap water (0.3 L/min) was applied to the wheel-rail contact, significantly less than the amount used in the previous study (7 L/min). The following conclusions were drawn from the obtained results:

1. Applying tap water to the wheel-rail contact can notably reduce the NC of both ultrafine and fine particles generated from the wheel-rail contact. Thus, the application of tap water proves to be a highly effective method to decrease the generation of AWPs from the wheel-rail contact.
2. In this study, the application of only 0.3 L/min of tap water on the wheel-rail contact resulted in a reduction of more than 67% and 86% of ultrafine and fine particles, respectively. Thus, even a small amount of applied tap water was found to be effective in reducing the generation of ultrafine and fine particles.
3. The presence of mineral crystals in tap water can influence the measured NC when tap water is used as a lubricant. The diffusion dryer employed in this study cannot eliminate mineral crystals. Therefore, a future study is necessary to investigate and verify the effect of mineral crystals on the experimental results.

## Figures and Tables

**Table 1:** Chemical composition and hardness of the disks.

Disk	Chemical Composition (wt. %)									Hardness (HB)
	Mn	C	Si	Cr	Cu	Ni	Mo	S	P	
Rail	0.79	0.63	0.25	0.13	0.15	0.06	0.01	0.12	0.21	291
Wheel										285

**Table 2:** Average reduction rates of ultrafine, fine, and total particles.

Train Velocity (km/h)	Reduction Rate (%)		
	Ultrafine Particles	Fine Particles	Total Particles
45	72.1	86.0	79.4
80	67.3	88.1	80.0

## Data Availability

Data is unavailable due to confidentiality.
